# Blockade of Lactate Dehydrogenase-A (LDH-A) Improves Efficacy of Anti-Programmed Cell Death-1 (PD-1) Therapy in Melanoma

**DOI:** 10.3390/cancers11040450

**Published:** 2019-03-29

**Authors:** Saeed Daneshmandi, Barbara Wegiel, Pankaj Seth

**Affiliations:** 1 Department of Medicine, Beth Israel Deaconess Medical Center, Harvard Medical School, Boston, MA 02215, USA; sdaneshm@bidmc.harvard.edu; 2 Division of Interdisciplinary Medicine, Beth Israel Deaconess Medical Center, Boston, MA 02215, USA; 3 Department of Surgery, Beth Israel Deaconess Medical Center, Harvard Medical School, Boston, MA 02215, USA; 4 Cancer Research Institute, Beth Israel Deaconess Medical Center, Harvard Medical School, Boston, MA 02215, USA

**Keywords:** LDH-A, lactate, PD-1, PD-L1, melanoma

## Abstract

Immunotherapy is a curable treatment for certain cancers, but it is still only effective in a small subset of patients. We have recently reported that programmed cell death protein-1 (PD-1) ligand (PD-L1) expression is regulated by lactate present at high levels in the tumor microenvironment (TME). We hypothesized that the efficacy of anti-PD-1 treatment can be improved by blocking the lactate-generating enzyme, lactate dehydrogenase-A (LDH-A). Anti-PD-1 treatment of mice harboring LDH-A deficient B16-F10 melanoma tumors led to an increase in anti-tumor immune responses compared to mice implanted with tumors expressing LDH-A. Specifically, we observed heightened infiltration of natural killer (NK) cells and CD8^+^ cytotoxic T cells in the LDH-A deficient tumors. These infiltrated cytotoxic cells had an elevated production of interferon-γ (IFN-γ) and granzyme B. Mechanistically, CD8^+^ T cells isolated from the TME of LDH-A deficient B16-F10 melanoma tumors and treated with anti-PD-1 showed enhanced mitochondrial activity and increased reactive oxygen species (ROS) levels. Moreover, infiltration of T regulatory (Treg) cells was diminished in LDH-A deficient tumors treated with anti-PD-1. These altered immune cell profiles were clinically relevant as they were accompanied by significantly reduced tumor growth. Our study suggests that blocking LDH-A in the tumor might improve the efficacy of anti-PD-1 therapy.

## 1. Introduction

Cancer cells rely on fermentative glycolysis to maintain their growth even under normoxic conditions, known as the Warburg effect [1]. Lactate dehydrogenase-A (LDH-A) is a key enzyme that catalyzes the last reaction of fermentative glycolysis, which converts pyruvate to lactate. LDH-A serves as a critical checkpoint within this pathway to regenerate nicotinamide adenine dinucleotide serves as a critical checkpoint within this pathway to regenerate nicotinamide adenine dinucleotide (NAD^+^). LDH-A levels are elevated in several cancer types and correlate with cancer growth, metastasis and recurrence as well as a poor clinical outcome [2,3]. We have recently demonstrated the role of LDH-A in myeloid cells in the tumor microenvironment (TME) as a driver of carcinogenesis [4]. Lactate, a metabolic product of LDH-A, is considered as an oncometabolite and elevated levels of lactate in cancer are associated with an increased number of metastases and lower survival. Recent studies indicate that lactate and/or low intra-tumoral pH promote an immunosuppressive TME with reduced infiltration of natural killer (NK) cells and cytotoxic CD8^+^ T lymphocytes and suppression of interferon-γ (IFN-γ) expression [5]. In vitro exposure to lactate and/or acidification of the culture medium suppress proliferation and cytokine production in human cytotoxic T lymphocytes (CTLs) and can be restored by increasing the pH [6]. Moreover, raising intra-tumoral pH with oral bicarbonate treatment can improve the anti-tumoral effect of immunotherapy in preclinical settings [7]. Together, these observations strongly suggest that decreasing lactate production in cancer cells, thereby preventing acidification of the TME, might synergize with immunotherapy. Recently, we reported that LDH-A-derived lactate within the TME upregulates expression of programmed cell death protein-1 (PD-1) ligand (PD-L1) on tumor cells [4]. The PD-1/PD-L1 pathway is one of the dominant immune checkpoints in the TME. Tumor cells express PD-L1, which suppresses T cell proliferation and effector function by ligating PD-1 expressed on activated T cells [8]. Beneficial effects of PD-1/PD-L1 blockade have been reported in several cancer types including breast cancer, melanoma, non-small cell lung, Hodgkin’s lymphoma, bladder, and kidney cancer [9]. However, the high efficacy of this treatment is limited to a subset of patients.

In this study, we investigated the role of LDH-A expressed in tumors for modulating immune responses to anti-PD-1 therapy in a B16-F10 melanoma model. We hypothesized that PD-1 blockade using an anti-PD-1 antibody might further inhibit the effects of the remaining PD-L1 expressed on tumor cells and block PD-1 signaling on immune cells. We demonstrate that deletion of LDH-A in melanoma cells abrogated PD-L1 expression and resulted in increased intra-tumoral immune cell infiltrates. These effects were assessed in the TME and were significantly enhanced by PD-1 blockade resulting in reduced tumor growth. These results also were confirmed by in vitro co-culture of CD8^+^ T cells with melanoma cells, resulting in enhancement of degranulation markers and mitochondrial function in the cultures of melanoma cells with the deletion of LDH-A with T cells.

## 2. Results

### 2.1. Treatment of Mice Harboring LDH-A Deficient B16-F10 Tumors with Anti-PD-1 Shows Reduced Tumor Growth, Lower Angiogenesis and Enhanced T Cell Infiltration

**Figure 1:**
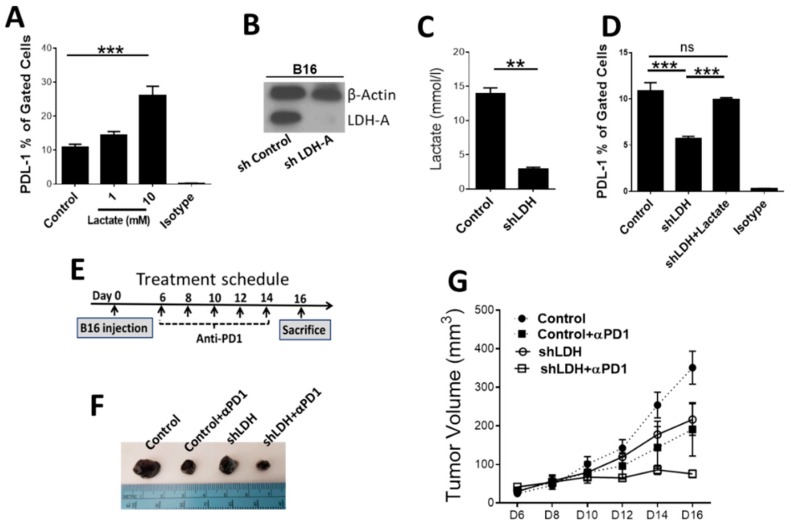
Lactate induces expression of programmed cell death protein-1 ligand (PD-L1) on B16 melanoma cells and deletion of lactate dehydrogenase-A (LDH-A) in B16 cells or anti-PD-1 therapy prevents tumor growth. (**A**) B16-F10 cells were treated with lactate (1 mM and 10 mM) for 24 h, and the level of PD-L1 was measured by flow cytometry. The number of PD-L1^+^ cells is shown as the percentage of gated cells. *** *p* < 0.001. (**B**) LDH-A was knocked down in B16-F10 melanoma cells using small hairpin RNA (shRNA) against LDH-A. Scramble shRNA was used as control. Western blotting with an antibody against LDH-A is shown on B16-green fluorescent protein (GFP) versus shLDH melanoma cells. (**C**) Capacity of lactate production was examined on B16-shLDH and control melanoma cells. ** *p* < 0.01. (**D**) B16-GFP and B16-shLDH melanoma cells were cultured in vitro and PD-L1 expression in the cells was measured by flow cytometry. *** *p* < 0.001, *n* = 3 independent experiments in triplicate. (**E**) Anti-PD-1 treatment schedule of mice tumor model. (**F**–**G**) Anti-PD-1 administration provided the same tumor growth inhibition as B16-shLDH tumor growth and anti-PD-1 injection induced additional tumor growth inhibition (*p* < 0.05). (**F**) demonstrates representative tumor size at day 16 after induction of tumor. ns: not significant.

We have previously reported that exogenous lactate increases PD-L1 expression in Lewis lung carcinoma (LLC) [4]. We demonstrate the same effect of exogenous lactate on melanoma cells (Figure 1A). We generated LDH-A deficient B16-F10 tumor cells (shLDH-A) by targeting LDH-A transcript with small hairpin RNA (shRNA). Control scramble shRNA was used to generate the appropriate control melanoma cell line. Low LDH-A protein levels in shLDH-A melanoma cells compared to control cells were confirmed by Western blot (Figure 1B and Supplementary Figure S1A). Similarly, diminished capacity for lactate production was confirmed in shLDH-A melanoma cells by colorimetric assay (Figure 1C). shLDH-A melanoma cells had significantly decreased PD-L1 expression compared to control cells (Figure 1D). Addition of exogenous lactate could override the effect of LDH-A deletion on the decrease of PD-L1 expression (Figure 1D) providing evidence for a direct causative link between lactate production and PD-L1 expression level. 

To determine the in vivo biological implications of our findings, we examined whether the shLDH-A B16-F10 melanoma cells had a different capacity to induce tumors in vivo compared to the control B16-F10 melanoma cells. shLDH-A and control B16-F10 melanoma cells were injected subcutaneously into syngeneic C57BL/6 mice and each group was divided into two subsets, which were treated either with the anti-PD-1 antibody or an isotype control. We confirmed the deletion of LDH-A in the tumors (Supplementary Figure S1B). Anti-PD-1 antibody treatment in recipients of control B16-F10 melanoma cells reduced tumor growth, consistent with previous observations [10]. Deletion of LDH-A in B16-F10 melanoma cells reduced tumor growth and treatment with anti-PD-1 antibody almost completely abrogated tumor growth in this group of mice (Figure 1F–G), suggesting an additive effect of both manipulations. Our immunostaining shows that shLDH-A deficient tumors were characterized by lower PD-L1 staining and had decreased proliferation and angiogenesis (Supplementary Figure S2).

### 2.2. Anti-PD-1 Therapy Induces Cytotoxic Responses in the TME of LDH-A Deficient B16-F10 Tumors

**Figure 2:**
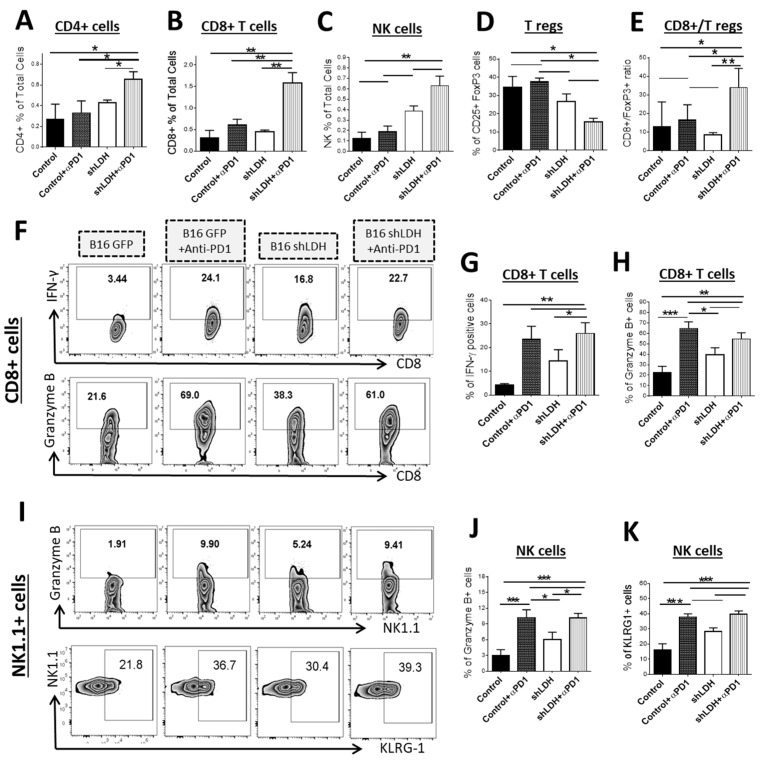
Immunophenotype of intra-tumoral immune cells. (**A–D**) Intra-tumoral infiltration of CD4^+^ T-cells (**A**), CD8^+^ T cells (**B**), natural killer (NK) cells (**C**), T regulatory (Treg) cells (**D**) and CD8^+^/Treg ratio (**E**) were evaluated by flow cytometry. The combination of anti-PD-1 use with the deletion of LDH-A in tumor cells induced high infiltration of these cytotoxic cells to the tumor microenvironment. * *p* < 0.05, ** *p* < 0.01. (**F**–**H**) Levels of granzyme B expression and IFN-γ production by intra-tumoral CD8^+^ T cells were determined using flow cytometry. Either application of anti-PD-1 or deletion of LDH-A in the tumor cells is correlated with higher levels of granzyme B or IFN-γ production by infiltrating CD8^+^ T cells at the tumor site. * *p* < 0.05, ** *p* < 0.01, *** *p* < 0.001. (**I**–**K**). Intra-tumoral infiltrating NK cells also express higher levels of granzyme B in the presence of anti-PD-1 or deletion of LDH-A in the tumor microenvironment. Intra-tumoral NK cells show elevated levels of KLRG-1 on their surface compared to control subjects. * *p* < 0.05, *** *p* < 0.001.

Inhibition of tumor growth by blockade of the PD-1/PD-L1 pathway correlates with the increase of T cell subsets in the TME [11]. To examine whether the observed difference in tumor growth in vivo was associated with distinct cell infiltrates, we examined fractions of CD4^+^ and CD8^+^ T cells, NK cells and T regularity (Treg) cells in shLDH-A deficient and control B16-F10 tumors (Figure 2). Fractions of CD4^+^ and CD8^+^ tumor infiltrating T cells were significantly increased after PD-1 antibody treatment only in recipients of shLDH-A deficient B16-F10 tumors. Notably, no significant increase in CD4^+^ and CD8^+^ tumor infiltrating T cells was observed between the PD-1 Ab-treated and isotype control-treated mice bearing LDH-A expressing B16-F10 tumors (Figure 2A,B). We showed a similar pattern using immunostaining with anti-CD8 antibodies in the TME (Supplementary Figure S3). We also observed similar pattern of changes in NK (NK1.1^+^) tumor infiltrating T cells in mice bearing shLDH-A and control B16-F10 tumors, treated with a PD-1 blocking (Figure 2C). The number of NK (NK1.1^+^) tumor infiltrating T cells was also increased in mice bearing shLDH-A B16-F10 tumors compared to those bearing control B16-F10 tumors even without treatment with the PD-1 blocking antibody (Figure 2C). This finding reveals the direct effect of lactate on the migration or expansion of NK T cells in the TME. 

An inverse profile was observed in the infiltrates of Treg cells in the TME, which were significantly decreased in the context of shLDH-A B16-F10 compared to control B16-F10 tumors even without anti-PD-1 antibody treatment. A lower number of Treg cells in this group was further enhanced by anti-PD-1 antibody treatment (Figure 2D). The calculation of the CD8^+^/Treg ratio demonstrates a higher trend in combination of LDH-A blocking and anti-PD-1 application (Figure 2E). We also detected an increased population of interleukin (IL)-17A^+^ CD8^+^ (TC17) cells in the spleens of mice after anti-PD-1 treatment (Supplementary Figure S4).

Effector T cells express IFN-γ and mediate cytolytic function through the key cytolytic mediators: perforin and granzyme B [12,13]. After CD8^+^ T cells recognize tumor cells, they release their cytolytic granules containing perforin and granzyme B to induce apoptosis in tumor cells [13]. Previous studies have shown that secretion of IFN-γ is an essential part of the mechanism of action of CD8^+^ tumor infiltrating lymphocytes (TIL) and blocking of IFN-γ production will suppress the anti-tumor activity of CD8^+^ T cells in the tumor microenvironment [12]. We examined IFN-γ-producing CD8^+^ cells and NK1.1^+^ cells infiltrating the tumor and found comparable fractions of IFN-γ producing cells in mice treated with the anti-PD-1 antibody regardless of the expression of LDH-A in B16-F10 tumors (Figure 2F, top panels). Importantly, mice bearing shLDH-A B16-F10 tumors had elevated IFN-γ-producing cell fractions even without PD-1 antibody treatment (Figure 2F, top panels). A similar profile was observed in granzyme B-expressing CD8^+^ and NK1.1^+^ T cells (Figure 2F, bottom panel, and Figure 2I, top panel).

Previous studies have reported that Killer Cell Lectin-Like Receptor Subfamily G positive (KLRG^+^) NK cells have strong protective function against metastatic tumors and high levels of lactate can reduce KLRG expression on NK cells as a mechanism for tumor cells to evade immune response [14]. We found that treatment with PD-1 blocking antibodies increased the fractions of KLRG-1^+^ NK1.1^+^ cells in the TME regardless of the expression of LDH-A in B16-F10 tumors. However, in mice bearing shLDH-A B16-F10 tumors, KLRG^+^ NK1.1^+^ cells were elevated even without PD-1 antibody treatment (Figure 2I, bottom panels). 

### 2.3. Infiltrating CD8^+^ CTLs Have Higher Metabolic Activation Status in an LDH-A Deficient Tumor Microenvironment

**Figure 3:**
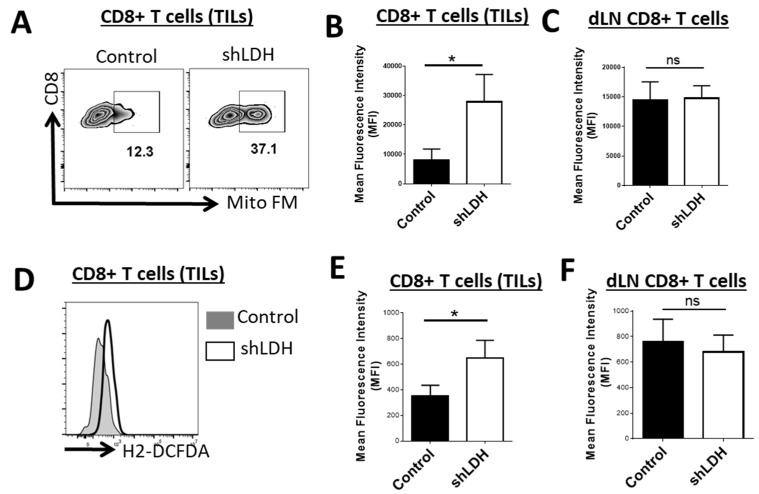
LDH-A deletion in tumor cells is associated with higher levels of mitochondrial mass and intracellular reactive oxygen species (ROS) production in infiltrating CD8^+^ T cells. (**A–C**) Representative flow cytogram of tumor-infiltrating CD8^+^ T cells (TILs) stained with MitoTracker FM and mean fluorescence intensity (MFI) (bar graph) of the cells from TILs or draining lymph node (dLN). (**D–F**) TILs or dLN CD8^+^ T cells were stained with the cellular ROS indicator 2′,7′-dichlorodihydrofluorescein diacetate (H2DCFDA). Average MFI (bar graph) demonstrates the higher capacity of CD8^+^ TILs to produce ROS in the LDH-A deficient tumor microenvironment while there is no significant difference in the dLN. *, *p* < 0.05.

Several studies have suggested that cancer cell-derived lactate modifies metabolism and subsequently the function of infiltrating immune cells [1,6]. We next evaluated the mitochondrial status of infiltrating CD8^+^ cells. CD8^+^ cells had increased levels of mitochondrial mass within their cytoplasm in LDH-A deficient tumors (Figure 3A,B) and this effect was TME-specific, as CD8^+^ T cells in draining lymph nodes did not show an enhanced number of mitochondria (Figure 3C). Scharping et al. have shown that suppression of mitochondrial mass and activity at the TME is a mechanism of immune evasion and drives intra-tumoral T cell metabolic insufficiency and dysfunction [15]. Therefore, improved mitochondrial function is beneficial foranti-tumor responses. Along with the increase of CD8^+^ T cell mitochondrial mass in LDH-A deficient tumors, infiltrating CD8^+^ T cells express higher levels of reactive oxygen species (ROS) (Figure 3D,E). The latter effect also is tumor site specific and was not seen in draining lymph nodes (Figure 3F). 

### 2.4. CD8^+^ T cells in In Vitro Co-Culture with LDH-A Deficient Tumor Cells Express Higher Levels of Degranulation Markers

**Figure 4:**
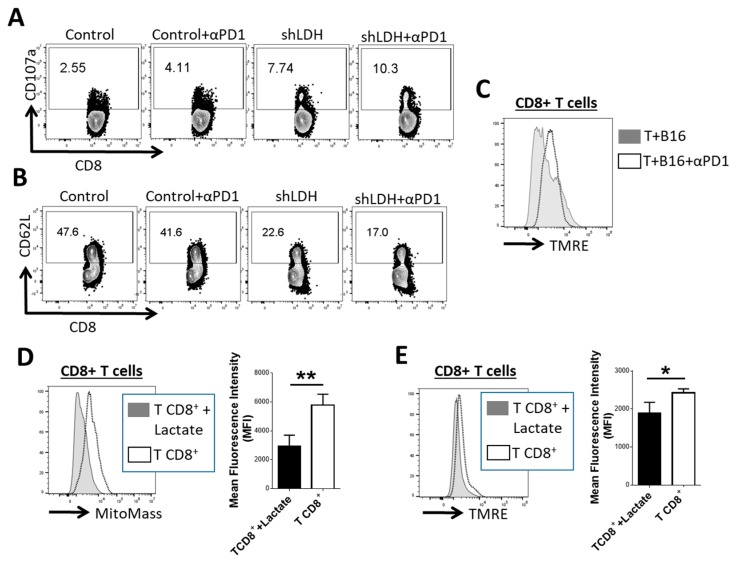
CD8^+^ T cells co-cultured with LDH-A deletion tumor cells or exposure to lactate in vitro. (**A**) Representative flow cytogram of a degranulation marker (CD107a) on CD8^+^ T cells, co-cultured for 24 h with LDH-A deleted melanoma cells in vitro. (**B**) Differential expression of CD62L marker on CD8^+^ T cells after co-culture for 24 h in vitro with LDH-A deleted B16-F10 melanoma cells. (**C**) Effect of added anti-PD-1 on CD8^+^ T cell’s mitochondrial potential (detected by tetra-methylrhodamine ester (TMRE)) while co-cultured with melanoma cells for 24 h; *p* < 0.05. (**D**–**E**) Mitochondrial mass and mitochondrial potential of CD8^+^ T cells exposed to l-lactate (10 mm) for 24 h. * *p* < 0.05. ** *p* < 0.01.

We evaluated CD8^+^ T cells co-cultured with melanoma cells to assess the specific responses of T cells in the presence or absence of LDH-A in the tumor cells. CD8^+^ T cells harvested from co-culture with shLDH-A melanoma cells demonstrated higher levels of CD107a expression (Figure 4A). CD107a also known as lysosomal-associated membrane protein-1 (LAMP-1) has been described as a substantial marker of CD8^+^ T-cell degranulation following stimulation [16]. CD8^+^ T cells cultured with shLDH-A melanoma also had lower expression of CD62L (Figure 4B). In both cases, the addition of anti-PD-1 further enhanced CD107a expression (Figure 4A) and diminished CD62L levels (Figure 4B). Addition of anti-PD-1 to the CD8^+^ cells: Melanoma co-culture led to higher activity of CD8^+^ T cell mitochondria compared to untreated cells (Figure 4C). We also exposed CD8^+^ T cells to 10 mM of lactate in vitro. After 24 h, CD8^+^ T cells treated with lactate had lower mitochondrial mass (Figure 4D) and lower mitochondrial potential (Figure 4E).

## 3. Discussion

The objective of this study was to investigate the role of the metabolic enzyme LDH-A in the modulation of immune anti-tumoral responses to anti-PD-1 immunotherapy. Several studies demonstrated that elevated levels of lactate in the TME due to metabolic switch (Warburg effect) results in the induction of an immunosuppressive milieu that facilitates the growth and invasiveness of tumors by altering the host immune system [1,5]. One of the key features of cancer resistance to therapy is the induction of co-inhibitory receptors such as checkpoint inhibitors [8]. We showed that melanoma tumor cells augmented the expression of PD-L1 after treatment with lactate, similarly to the lung cancer model [4]. Moreover, blocking of LDH-A in melanoma cells via shRNA prevented the enhancement of PD-L1 expression. Suppression of PD-L1 expression by blockade of LDH-A in melanoma cells was validated in vivo in an LDH-A deficient B16 melanoma tumor model. This enhanced PD-L1 expression would be correlated with metabolic changes in TME.

The rapid growth of tumor cells creates a hypoxic environment, resulting in enhanced glycolytic metabolism and increased lactate in the TME. Lack of LDH-A in myeloid cells resulted in lower hypoxia-inducible factor 1-alpha (HIF1α) expression, suggesting a potential mechanism of PD-L1 regulation in response to the LDH-A/lactate axis [4]. HIF1α has shown to be a target of lactate in cancer cells [17]. Other studies have also shown that hypoxia mediated through HIF1α regulates expression of PD-L1 [18]. Moreover, studies using human lung cancer models suggested alteration of PD-L1 by lactate through G protein-coupled receptor 81(GPR81) in a TAZ-dependent pathway [19]. Recently, another group highlighted pyruvate, an upstream metabolite of lactate, as a regulator of PD-L1 expression via induction of the bone morphogenetic protein 4/phosphorylated SMAD1/5/IFN regulatory factor 1(1BMP4/p-SMAD1/5/IRF1) signaling pathways [20]. 

Our results show that LDH-A-deficient tumors are significantly more responsive to anti-PD-1 treatment compared to control tumors expressing LDH-A. These results are in line with previous studies on advanced/metastatic melanoma patients treated with anti-PD-1. This study demonstrated that patients with an elevated baseline LDH-A had significantly shorter survival and elevation of serum LDH-A correlated with progressive disease [21]. Blocking of LDH-A in tumor cells effectively enhances infiltration of CD8^+^ T cells and NK cytotoxic cells in the TME. These infiltrating NK cells and CD8^+^ cytolytic cells expressed higher amounts of granzyme B in the TME of tumors in mice treated with anti-PD-1 therapy. Moreover, intra-tumoral NK cells had elevated levels of KLRG-1 on their surface. KLRG-1 is a marker of terminally differentiated NK cells and T cells that binds to several members of the cadherin family. KLRG-1 is considered as an inhibitory receptor. However, the anti-tumoral function of KLRG-1^+^ NK cells in the TME was indicated in pulmonary metastatic disease [14]. Increased expression of PD-1 on NK cells prevents NK cell-mediated anti-tumor function and is correlated with poor prognosis in digestive cancers [22]. It has been well documented that lactate in the TME induces immunosuppression, which results in T cell and NK cell apoptosis and reduces the frequency of infiltrates into these tumors [23]. 

In this study, we have examined metabolic alterations of infiltrating CD8^+^ T cells in our treatment groups. Intra-tumoral CD8^+^ T cells demonstrated higher amounts of mitochondrial mass and ROS production accompanied by an increased capacity of IFN-γ production in LDH-A deficient tumors. Previous in vitro studies implied that lactate secreted by tumor cells could alter CD8^+^ T cell function and that these functions can be reversed by incubation in lactic acid-free medium for 24 h [6]. We also showed lower mitochondrial function in response to lactate and higher mitochondrial mass in CD8^+^ T cells treated with anti-PD-1 in co-culture with melanoma cells in vitro. LDH-A deficient tumors facilitate oxidative phosphorylation by shuttling pyruvate into mitochondrial metabolism, resulting in higher production of ROS [24]. Increased levels of ROS are also directly linked to higher mitochondrial biomass in CD8^+^ T cells in LDH-A deficient tumors. Prior studies showed the importance of mitochondrial biogenesis during early effector maturation of CD8^+^ T cells [25]. However, abrogation of mitochondria-derived ROS in PD-1^+^ CD8^+^ TILs by a protein kinase R-like endoplasmic reticulum kinase (PERK) inhibitor bolstered CD8^+^ TIL viability [26]. 

Lactic acid from melanoma cells inhibited tumor associated antigen (TAA)-triggered IFN-γ secretion in three-dimensional (3D) melanoma spheroid co-cultures [27]. Mendler et al. similarly confirmed that lactic acidosis reduced T cell receptor (TCR)-driven IFN-γ, tumor necrosis factor-alpha (TNF-α) and IL-2 expression and CTL functions, suggesting MAPKs p38 and JNK/c-Jun signaling pathways as the mechanism of suppression [28]. Another study reported that lactic acid and acidification diminish nuclear factor of activated T-cells (NFAT) levels resulting in diminished IFN-γ production and prevent T cell and NK cell activation [23]. 

Therefore, blocking of LDH-A and PD-1 might shift the tumor milieu towards pro-inflammatory and anti-tumor responses due to the activation of mitochondria and ROS-mediated signaling. Indeed, increased ROS levels in the TME enhanced IFN-γ-induced apoptosis of colon cancer cells [29]. However, the levels of ROS implicated in tumor suppression versus promotion are still under debate [30]. 

Altogether, our results demonstrate higher effectiveness of anti-PD-1 treatment after blockade of LDH-A in melanoma. Such combination treatment strongly blocks the PD-1/PD-L1 pathway, leading to elevation of pro-inflammatory anti-tumor responses such as higher infiltration and activity of NK cells and CD8^+^ cytotoxic cells and a diminished frequency of Treg cells. Our results indicate that mice with LDH-A deficient tumors treated with anti-PD-1 have low growth due to the additive effects of reduced lactate and decreased tumoral PD-L1 expression which itself is regulated by lactate. Current LDH-A inhibitors are not optimal for in vivo therapies, yet the fast progress of immunometabolic targets and therapies may lead to better strategies to target LDH-A pharmaceutically. Based on our results, we suggest that modulation of LDH-A/lactate could regulate PD-L1 expression and be an effective way to improve the efficacy of anti-PD-1 therapy. 

## 4. Materials and Methods

### 4.1. Mice

Female C57BL/6 mice (6–8 weeks old) were purchased from Jackson Laboratories. All animals were held under specific pathogen-free conditions and humanely euthanized by CO_2_ inhalation according to the standard protocols, which were approved by the Institutional Animal Care and Use Committee (IACUC) at Beth Israel Deaconess Medical Center (Boston, MA) (040-2016, approved 18 August 2018). 

### 4.2. B16-F10 Melanoma Cell Line and Culture

B16-F10 melanoma cells (obtained from American Type Culture Collection (ATCC) in 2013) were in stored at passage 3 (used in experiments in up to passage 10), tested for mycoplasma in 2015, and cultured in complete media (CM): Dulbecco’s Modified Eagle Medium (DMEM) media supplemented with 10% heat-inactivated fetal bovine serum (FBS), l-glutamine, 100 mg/mL streptomycin and 100 U/mL penicillin. LDH-A was knocked down using shRNA against LDH-A. Scramble shRNA was used as control. To examine the effect of lactate in vitro, B16-F10 melanoma cells (5 × 10^5^ cell/mL) were cultured in RPMI media with FBS and treated with l-lactate (1 mmol/L and 10 mmol/L) or control for 24 h. After incubation with l-lactate, PD-L1 expression was examined by flow cytometry using anti-PD-L1 (clone 10F.9G2, BioLegend, Cambridge, MA, USA). Lactate release from the cultured cells was determined by Lactate Colorimetric Assay Kit II (BioVision Inc., Milpitas, CA, USA). 

CD8^+^ T cells were isolated from the spleen and lymph nodes of C57BL/6 mice using negative selection on magnetic beads (MACS, STEMCELL Technologies, Cambridge, MA, USA) and cultured in presence of 10 mM l-lactate for 24 h at 37 °C. In another set of experiments, purified CD8^+^ T cells were co-cultured with melanoma cells (1:1 ratio) for 24 h and then degranulation capacity (determined by CD107a staining) and mitochondrial activity were detected.

### 4.3. Tumor Model and Anti-PD-1 Treatment

To establish the tumor model, 2 × 10^5^ of either B16-GFP or B16-shLDH melanoma cells were implanted in to the left flank of C57BL/6 mice. We randomized mice into two experimental arms: (i) treated with anti-PD-1 antibody (clone 29F.1A12, 200 µg, i.p.) and (ii) control mice injected with of isotype control (200 µg, i.p.) every two days. Anti-PD-1 treatment was applied seven days after cancer cell implantation when the tumors were palpable. Tumor diameters were measured with a digital caliper every two days and tumor volume in mm^3^ was calculated by the formula: volume = (width)^2^ × length/2. 

### 4.4. Flow Cytometry

After harvesting and washing with 1× PBS, cells were stained with PerCP anti-CD3 (clone 145-2C11, BioLegend, Cambridge, MA, USA), PE anti-mouse CD4 (clone 4B12, Biolegend), FITC anti-CD8 (clone 53-6.7, BioLegend), APC anti-PD-L1 (clone 10F.9G2, BioLegend), PE/Cy7 anti-CD25 (clone PC61, BioLegend), PE anti-FoxP3 (clone 150D, BioLegend), Pacific Blue anti-NK1.1 (clone PK136, BioLegend), APC anti-Granzyme B (clone QA16A02, BioLegend), APC/Cy7 anti-CD107a (clone 1D4B, BioLegend), APC/Cy7 anti-KLRG-1 (clone 2F1/KLRG-1, BioLegend), APC anti-IFN-γ (clone XMG1.2, BioLegend). After staining, cells were analyzed by a CytoFLEX flow cytometer (Beckman Coulter, Indianapolis, IN, USA) and then percentage of gated cells was calculated using FlowJo_V10 software (FlowJo, LLC, Ashland, OR, USA).

### 4.5. Mitochondrial Mass, Mitochondrial Poetical and Reactive Oxygen Species Production

Cells were cultured in flow cytometry (FACS) staining buffer (PBS containing 2% FBS) and incubated with 0.2 µM of MitoTracker Deep Red FM (Invitrogen, Eugene, OR, USA) for 30 min at 37 °C. Mitochondrial potential (ΔΨm) was detected by adding 200 nM tetra-methylrhodamine ester (TMRE) (Thermo Fisher, Eugene, OR, USA). For ROS detection, the cells in FACS buffer were treated with 20 μM 2′,7′-dichlorodihydrofluorescein diacetate (H2DCFDA; Invitrogen,) for 30 min at 37 °C. Cells then were washed with FACS buffer and analyzed by a CytoFLEX flow cytometer (Beckman Coulter, Indianapolis, IN, USA).

### 4.6. Immunostaining

Tumor tissues were isolated and frozen in freezing medium using ice-cold methyl butane. Tissues were cut in 6 µm sections using a CryoTome (Fisher Scientific, Loughborough, UK); sections were placed on glass slides, then stored at −80 °C or immediately used for staining. Tissue sections were then fixed with 2% paraformaldehyde (PFA) followed by permeabilization with 0.5% Triton X-100. After blocking with 7% horse serum (Vector Laboratories, Burlingame, CA, USA) in PBS, primary antibodies were then applied overnight at 4 °C. Sections were then incubated with fluorescently labeled secondary antibodies for 1 h at room temperature. The images were captured using a Fluorescence Microscope (Zeiss, Thornwood, NY, USA). The following antibodies were used: anti-CD8 (BioLegend), anti-PD-L1 (BioLegend), anti-Phospho-Histone H3 (P-HH3) (Cell Signaling) and anti-CD31 (BD Biosciences).

### 4.7. Western Blotting

For Western blotting, the cells were lysed on ice in modified Radioimmunoprecipitation assay (RIPA) buffer (50 mmol/L TrisHCl (pH 7.4), 50 mmol/L sodium fluoride, 150 mmol/L NaCl, 1% Nonident P40, 0.5 mol/L EDTA (pH 8.0)) supplemented with Complete Proteinase Inhibitor Cocktail (Roche, Mannheim, Germany). Supernatants of the samples were collected after centrifugation at 14,000× *g* and protein concentration was determined using a Bicinchoninic Acid Protein Assay Kit (ThermoFisher, Rockfold, IL, USA). Protein (20 μg of each sample) was electrophoresed on 4–12% Bis-Tris Gel (Life Technologies, Carlsbad, CA, USA) and transferred to the membrane. The membranes were blocked with 5% non-fat milk in 1× Tris-Buffered Saline (TBS) (Boston BioProducts, Ashland, MA, USA) for 1 h and probed with primary antibodies overnight. Membranes were washed with TBS and visualized using Super Signal West Pico chemiluminescent substrate (ThermoFisher). The following antibodies were used: anti-LDH-A (Abcam, Eugene, OR, USA) and anti-β-actin (Sigma Aldrich, Burlington, MA, USA).

### 4.8. Statistical Analysis

All data are presented as mean ± standard deviation (SD) unless otherwise indicated. Comparison between groups was performed using the Student *t*-test or one-way analysis of variance (ANOVA), followed by the post-hoc Tukey test. All statistical evaluations were performed using Graph Pad Prism (San Diego, CA, USA) and *p*-values < 0.05 considered statistically significant. 

## Supplementary Materials

The following are available online at https://www.mdpi.com/2072-6694/11/4/450/s1, Figure S1: LDH-A Western Blot, Figure S2: Intra-tumor staining, Figure S3: Intra-tumor CD8^+^ infiltration, Figure S4: TC17 frequency.
