# Supplementary material for: Blockade of Lactate Dehydrogenase-A (LDH-A) Improves Efficacy of Anti-Programmed Cell Death-1 (PD-1) Therapy in Melanoma
Source: Cancers (Basel). 2019 Mar 29;11(4):450. doi: 10.3390/cancers11040450 (PMC6521327; doi:10.3390/cancers11040450)
Supplement: Supplementary file 1 [file cancers-11-00450-s001.pdf]

Supplementary Information:

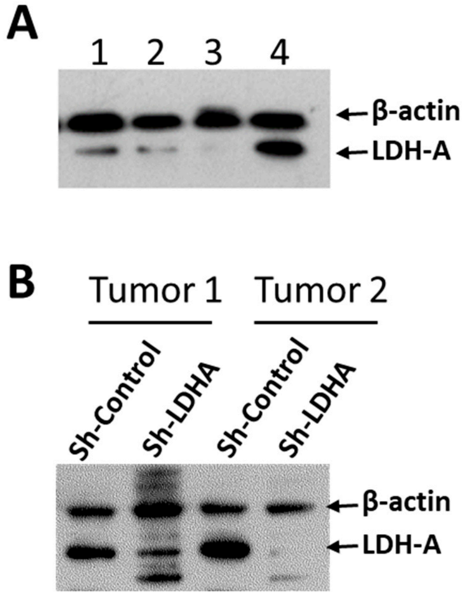

Figure S1. Western blotting with antibody against LDH-A is shown on B16-GFP versus shLDH melanoma cells. (**A**) In cell line before treatment. 1: B16F10 experiment 1 + shLDH; 2: B16F10 experiment 2 + shLDH; 3: B16F10 experiment 3 + shLDH; 4: B16F10 experiment 4 + Scramble shRNA. β-Actin as control. Cell line 3 was used as shLDH and cell line 4 was used as control in the subsequent experiments. (**B**) In implanted tumors.

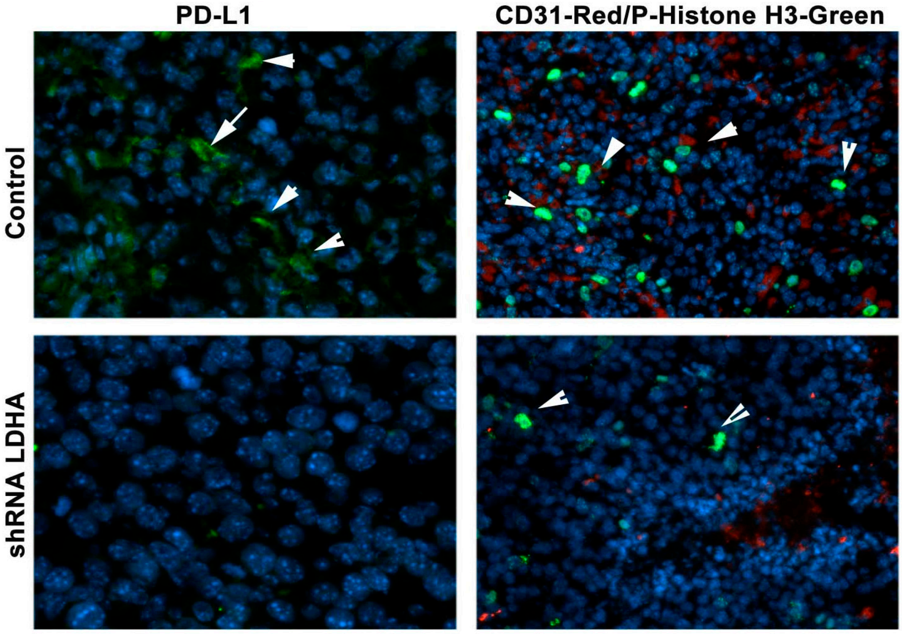

Figure S2. Intra-tumoral PD-L1, CD31 and P-HH3 expressions. A. Immunofluorescence staining with antibodies against PD-L1 on tumor tissues from B16 or B16-sh. Green: PD-L1; Blue: melanoma cell nuclei. (*n* = 2 to 3 per group). Analysis of tumor tissue slices by IFC for angiogenesis (indicated as CD31 expression) and proliferation (P-Histone H3). Immunofluorescence staining with antibodies against CD31 and histone H3 on tumor tissues from B16 or B16-sh (*n* = 2 to 3 per group).

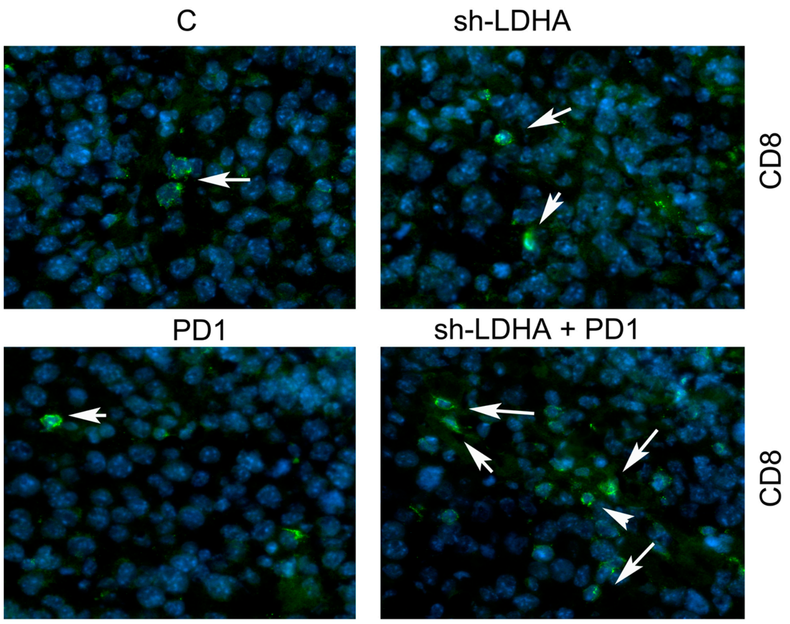

Figure S3. Intra-tumoral infiltration of CD8$^+$ T cells Immunofluorescence staining with antibodies against CD8 on tumor tissues from B16 or B16-sh treated or untreated with anti-PD-1.

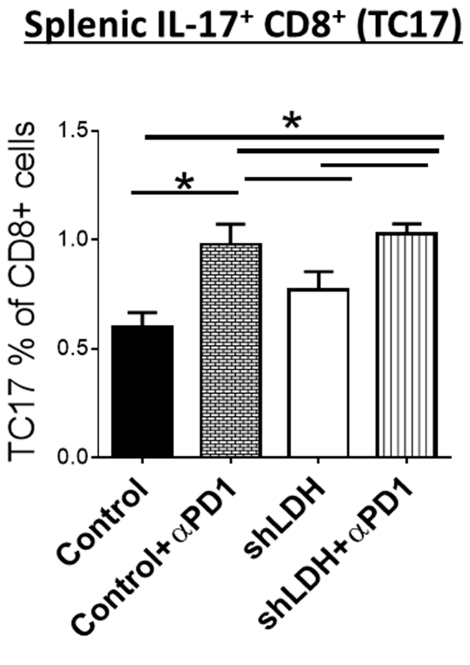

Supplementary Figure 4. Increased population of IL-17A$^+$ CD8$^+$ (TC17) cells in mice spleen after anti-PD1 treatment. Splenic IL-17A$^+$ CD8$^+$ (TC17) cells frequency in different treatment groups.
